# Distribution of Vaginal and Gut Microbiome in Advanced Maternal Age

**DOI:** 10.3389/fcimb.2022.819802

**Published:** 2022-05-27

**Authors:** Yuxin Huang, Dianjie Li, Wei Cai, Honglei Zhu, Mc Intyre Shane, Can Liao, Shilei Pan

**Affiliations:** ^1^Department of Gynaecology and Obstetrics, Zhujiang Hospital, Southern Medical University, Guangzhou, China; ^2^The First School of Clinical Medicine, Southern Medical University, Guangzhou, China; ^3^The Second School of Clinical Medicine, Southern Medical University, Guangzhou, China; ^4^Prenatal Diagnostic Center, Guangzhou Women and Children’s Medical Center, Guangzhou, China

**Keywords:** advanced maternal age, vaginal microbiota, gut microbiota, diversity, abundance

## Abstract

The distribution of the microbiome in women with advanced maternal age (AMA) is poorly understood. To gain insight into this, the vaginal and gut microbiota of 62 women were sampled and sequenced using the 16S rRNA technique. These women were divided into three groups, namely, the AMA (age ≥ 35 years, *n* = 13) group, the non-advanced maternal age (NMA) (age < 35 years, *n* = 38) group, and the control group (non-pregnant healthy women, age >35 years, *n* = 11). We found that the alpha diversity of vaginal microbiota in the AMA group significantly increased. However, the beta diversity significantly decreased in the AMA group compared with the control group. There was no significant difference in the diversity of gut microbiota among the three groups. The distributions of microbiota were significantly different among AMA, NMA, and control groups. In vaginal microbiota, the abundance of *Lactobacillus* was higher in the pregnant groups. *Bifidobacterium* was significantly enriched in the AMA group. In gut microbiota, *Prevotella bivia* was significantly enriched in the AMA group. Vaginal and gut microbiota in women with AMA were noticeably different from the NMA and non-pregnant women, and this phenomenon is probably related to the increased risk of complications in women with AMA.

## Introduction

The International Federation of Gynecology and Obstetrics refers to maternal age ≥35 years at the time of expected delivery as advanced maternal age (AMA). The proportion of births to women of AMA has increased over time throughout the developed world and in developing countries because of improved awareness and availability of effective contraception and improvement in assisted reproductive technology (Hsieh et al., 2010; Matthews and Hamilton, 2014; Arya et al., 2018). According to a survey in 2011, the proportion of AMA was 10.1%, including 8.3% aged 35–39 and 1.8% aged 40 or older in China (Liu et al., 2015). Women with AMA are also at increased risk of adverse pregnancy outcomes, such as gestational diabetes mellitus, hypertensive disorders of pregnancy, preterm delivery, fetal dysplasia, and fetal growth restriction (Cakmak et al., 2017; Fuchs et al., 2018; Marozio et al., 2019; Teng et al., 2020).

Pregnancy is a special physiological process in women. The microecology of different body sites will change during pregnancy as the hormones, immunity, metabolism, and other changes occur in women. Several differences in vaginal microecology occur during pregnancy in women of different ages (Li and Wang, 2020). Age is also a factor affecting the composition of oral microbiota in pregnant women (Wang T. et al., 2021). The diversity of gut microbiota decreases significantly in women with AMA and gestational diabetes (Huang, 2019). In addition, the colonization and diversity of gut microbiota are influenced by internal and external environmental factors during the co-evolution of the gut microbiome with their hosts, such as gender, age, race, dietary characteristics of the hosts, genotype, immune system, and region (Gohir et al., 2015b; Winer et al., 2016; Kvit and Kharchenko, 2017). It is necessary to understand the vaginal and gut microbiome in women with AMA. Therefore, we propose a hypothesis that the microbiota in women with AMA may be dysregulated. In order to provide direction for the prevention of adverse pregnancy outcome of women with AMA, we need to explore the specific bacteria of women with AMA. To confirm our hypothesis and explore the correlation between the microbiome and advanced pregnancy, we tested the distribution of vaginal and gut microbiota in women with AMA during the third trimester using 16S rRNA gene sequencing in this research.

## Methods

### Study Population and Sampling

A total of 51 healthy pregnant women who had antenatal care at Zhujiang Hospital, Southern Medical University were recruited from May 2020 to August 2020. They were divided into the AMA (age ≥ 35 years, *n* = 13) and non-advanced maternal age (NMA) (age < 35 years, *n* = 38) groups based on the age at the expected date of delivery. All of them were singleton pregnancies. A number of 11 non-pregnant healthy women over 35 years old were recruited as the control group. Women experiencing or undergoing the following were excluded: hematochezia, vaginal bleeding, sexual intercourse, rigorous cleaning with or without the use of any cleaning or disinfecting agents in the vaginal or perianal area within 7 days of sampling, treatment with antibiotics within 30 days of sampling, prenatal bleeding, premature rupture of membranes, placenta previa, placental abruption, or previa blood vessels.

We collected vaginal and rectal swabs from non-pregnant healthy women who were not menstruating and women between the 36th and 37th week of gestation in AMA and NMA groups. Vaginal secretions were sampled from the lower third of the vagina and feces were sampled from the rectum using a sterile swab. Meanwhile, vaginal and gut secretions were collected in the same swab for every pregnant women to culture whether they were infected with group B streptococcus. Moreover, the blank control group included three swabs, namely, a blank sample swab, a swab with the laboratory air sample, and a swab with the laboratory distilled water sample. All swabs were placed on ice immediately before being snap-frozen and stored at −80°C within 5 min of collection until DNA extraction.

Similar to Wang’s study (Wang Y. et al., 2021), a self-designed questionnaire by us was also used to evaluate the past year frequency and structure of maternal diet prior to delivery. As recommended by the Chinese Society of Nutrition (Zeng, 2018), six common foods for Chinese women during pregnancy were collected, namely, rice, flour, meat, vegetables, fruit, and yogurt. We assessed the intake of these foods every day.

The information of women was obtained from medical records. Neonatal information and histology of the placentas were collected after delivery. The histology of the placentas included normal placenta and mild, moderate, or severe fetal membrane inflammation. This study was carried out with the approval of the Ethics Service Committee of Zhujiang Hospital, Southern Medical University (China).

### Total Bacterial DNA and 16S rRNA Gene Sequencing

We extracted the total bacterial DNA from vaginal secretions and feces by a hexadecyltrimethylammonium bromide (CTAB) and magnetic soil and stool DNA kit. The purity and concentration of the DNA were monitored on 1% agarose gel electrophoresis. We used genome DNA that had been diluted with sterile water to 1 ng/μl as a template to perform polymerase chain reaction (PCR). To identify the bacterial diversity, we used the barcoded 515F 5′-CCTAYGGGRBGCASCAG-3′ and 806R 5′-GGACTACNNGGGTATCTAAT-3′ primers to amplify bacterial 16S rRNA V3–V4 fragments. Thermal cycling consisted of initial denaturation at 98°C for 1 min, followed by 30 cycles of denaturation at 98°C for 10 s, annealing at 50°C for 30 s, elongation at 72°C for 30 s, and finally 72°C for 5 min. The same volume of 1X loading buffer (contained SYB green) was mixed with PCR products and electrophoresis was carried out on 2% agarose gel for detection. PCR products were mixed in equidensity ratios. Then, the mixture of PCR products was purified with a Qiagen Gel Extraction Kit (Qiagen, Germany). Sequencing libraries were generated using the TruSeq^®^ DNA PCR-Free Sample Preparation Kit (Illumina, USA) following the manufacturer’s recommendations and index codes were added. The library quality was assessed on the Qubit@ 2.0 Fluorometer (Thermo Scientific) and the Agilent Bioanalyzer 2100 system. Lastly, the library was sequenced on an Illumina NovaSeq platform and 250-bp paired-end reads were generated.

### Data Analysis

Paired-end reads were assigned to samples based on their unique barcode and truncated by cutting off the barcode and primer sequence. Paired-end reads were merged using FLASH (Magoc and Salzberg, 2011), a very fast and accurate analysis tool, which was designed to merge paired-end reads when at least some of the reads overlap the read generated from the opposite end of the same DNA fragment, and the splicing sequences were called raw tags. Quality filtering on the raw tags was performed under specific filtering conditions to obtain high-quality clean tags (Bokulich et al., 2013) according to the QIIME (Caporaso et al., 2010) quality-controlled process. The tags were compared with the Silva database using the UCHIME algorithm (Edgar et al., 2011) to detect chimera sequences, and then the chimera sequences were removed (Haas et al., 2011). Then, the effective tags were finally obtained.

Alpha diversity is applied in analyzing the complexity of species diversity of a sample through three indices, namely, the Shannon index, phylogenetic diversity (PD), and abundance-based coverage estimator (ACE). The Shannon index considers the total number of species and their relative abundances. PD is based on the proportion of branch length in a phylogenetic tree that leads to different organisms, whereas ACE is used to estimate the total number of species contained in a community sample. All these indices in our samples were calculated with QIIME (Version 1.9.1) and displayed with R software (Version 2.15.3).

Beta diversity analysis was used to evaluate differences of samples in species complexity. Beta diversity on both weighted and unweighted UniFrac distances were calculated by QIIME (Version 1.9.1). Principal coordinate analysis (PCoA) was performed to obtain principal coordinates and visualize complex, multidimensional data. A distance matrix of weighted or unweighted UniFrac distances among samples obtained before was transformed into a new set of orthogonal axes, by which the maximum variation factor is demonstrated by the first principal coordinate, the second maximum variation factor by the second principal coordinate, and so on. PCoA was displayed by the WGCNA package, stat packages, and the ggplot2 package in R software (Version 2.15.3). UniFrac evaluates microbiota similarity based on the shared evolutionary history of bacterial taxa (Lozupone and Knight, 2005) but does not control subject covariate information.

Linear discriminant analysis effect size (LEfSe) (Segata et al., 2011) is a tool for discovering and interpreting biomarkers and emphasizes statistical significance and biological correlation. It can search for statistically different biomarkers between groups.

### Statistical Analysis

The social characteristics of participants and diet were analyzed based on *t*-tests, performed in IBM SPSS (Statistical Package for the Social Sciences, version 21.0) at a 5% level of significance. The measurement data were tested for normality using the Kolmogorov–Smirnov test first. If the data conformed to normal distribution, they were expressed as mean ± standard deviation (x ± s) and analyzed with *t*-test. If the data were not normally distributed, the median was used for expression and the Mann–Whitney test was used for comparison. *t*-test and Wilcoxon tests were used for the analyses of alpha and beta diversity indexes between groups. Two-tailed *p*-values <0.05 were considered statistically significant unless otherwise stated.

## Results

### Characteristics of the Study Population

The diets of women consisted mainly of grains, vegetables, and meat in the AMA and NMA groups (**Table 1**). No significant differences were observed with regard to pre-pregnancy body mass index (BMI), mode of delivery, gestational age of sample collection and delivery, gender of newborns, or weight and height of newborns between the AMA and NMA groups (*p* > 0.05). Gravidity and parity in the AMA group were higher than those in the NMA group (*p* < 0.05) (**Table 1**). No women were infected with group B streptococcus in the two groups. No difference was observed in the histology of placentas between the two groups (*Z* = −1.08, *p* = 0.28).

**Table 1:** Clinical and anthropometric characteristics of the mothers and their newborns.

Characteristics	AMA group (*n* = 13)	NMA group (*n* = 38)	*p*-value
Age (years)	36.46 ± 1.81	29.42 ± 3.01	0.000*
Gravidity	3.15 ± 1.57	1.89 ± 1.03	0.002*
Parity	2.15 ± 0.80	1.53 ± 0.69	0.009*
Progestational BMI (kg/m^2^)	18.94 ± 6.43	20.22 ± 1.87	0.510
Gestational age of sample collection (weeks)	35.21 ± 1.12	34.39 ± 1.58	0.094
Gestational age of sample collection (weeks)	39.03 ± 1.11	39.46 ± 0.84	0.160
Mode of delivery			0.027*
Vaginal delivery	6	27	
Cesarean section	7	7	
Gender of newborns			0.282
Male	7	15	
Female	6	9	
Weight of newborns (kg)	3.33 ± 0.42	3.25 ± 0.30	0.506
Height of newborns (cm)	49.77 ± 1.69	50.15 ± 1.52	0.461
Daily intake (g)			
Rice	207.69 ± 53.41	192.11 ± 43.51	0.298
Flour	37.69 ± 14.23	37.89 ± 31.03	0.982
Meat	155.38 ± 30.44	148.95 ± 39.78	0.598
Vegetables	359.23 ± 77.94	350.53 ± 75.19	0.723
Fruits	149.23 ± 46.99	171.84 ± 50.29	0.161
Yogurt	61.54 ± 46.34	70.53 ± 54.57	0.598

*The difference was significant.

### Diversity of Vaginal and Gut Microbiota

DNA extraction, PCR amplification, and electrophoretic detection in all the samples were deemed successful except for the samples in the blank control group.

**Figure 1:**
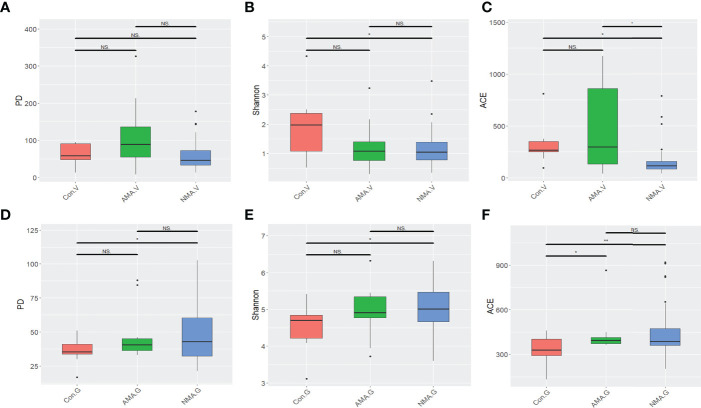
**(A–C)** Alpha diversity index differences in vaginal microbiota between groups (Wilcoxon test). **(D–F)** Alpha diversity index differences in gut microbiota between groups (Wilcoxon test). NS, not statistically significant; *p < 0.05; **p < 0.01.

In terms of the three alpha diversity measures in vaginal microbiota, we observed that the index of PD and ACE in AMA was obviously higher than that in NMA (**Figures 1A–C**). The AMA group had a significantly higher alpha diversity than the NMA group (ACE *p* < 0.05). More species were detected in the vaginal microbiota of the AMA group, and the community diversity was higher. On the contrary, the Shannon index was lower, and PD and ACE were higher in the AMA group than in the control group. The Shannon index indicates the total number of taxa in the sample and their percentage. The higher the community diversity and the more even the species distribution, the higher the Shannon index will be. Therefore, the distribution of species in the AMA group was more uneven than that in the control group. As for gut microbiota, the alpha diversity of the AMA group was slightly lower than that of the NMA group, but significantly higher than that of the control group (ACE *p* < 0.05) (**Figures 1D–F**). The community diversity was lower, and distribution was uneven in the gut microbiota of the AMA group, whereas all differences in the gut microbiota between the AMA and NMA groups were not statistically significant (*p* > 0.05).

**Figure 2:**
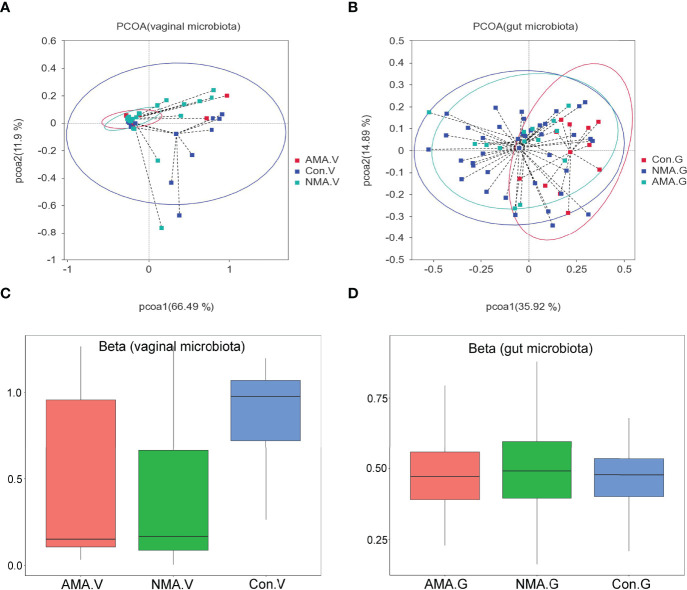
**(A, B)** PCoA (principal coordinates analysis) based on weighted UniFrac distance. **(C, D)** Beta diversity index differences between groups (Wilcoxon test). The abscissa represents a principal component, the ordinate represents another. The percentage represents the contribution value of the principal component to the sample difference. Each dot represents a sample, and samples in the same group are indicated in the same color.

PCoA was performed using weighted UniFrac distance analysis to cluster the three groups at the OTU levels. We found that PCoA clearly separated patients in the control group and pregnant groups in terms of vaginal and gut microbiota. However, the distance between the AMA and NMA groups was close, which implies that the compositions of the microbiomes were similar (**Figures 2A, B**). The median and mean of the AMA and NMA groups were approximate. Thus, no difference in vaginal and gut microbiota was observed between the two groups (**Figure 2C**). The beta diversity of the vaginal microbiota in the AMA group significantly decreased compared with the control group (*p* < 0.001), whereas no difference in gut microbiota was noticed between the two groups (**Figure 2D**).

### Distribution of Vaginal and Gut Microbiota

**Figure 3:**
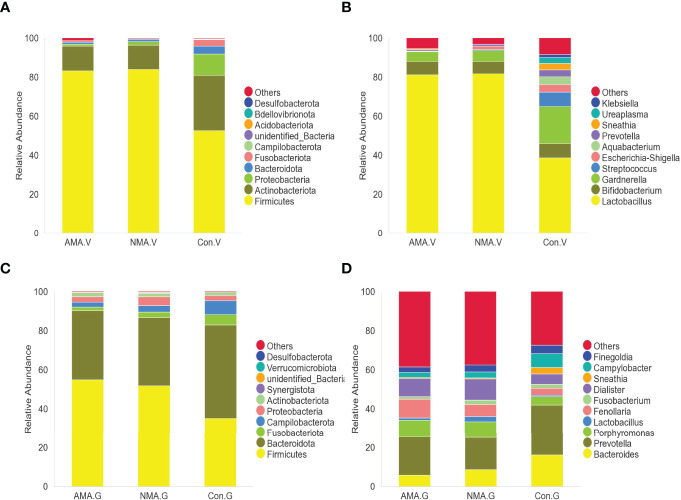
**(A)** Abundance of species at the phylum level in vaginal microbiota. **(B)** Abundance of species at the genus level in vaginal microbiota. **(C)** Abundance of species at the phylum level in gut microbiota. **(D)** Abundance of species at the genus level in gut microbiota.

The vaginal microbiota of the three groups mainly included *Firmicutes*, *Actinobacteriota*, *Proteobacteria*, and *Bacteroidota*. *Firmicutes* were abundant in pregnant women, with a proportion of 83.4% in the AMA group and 84.1% in the NMA group, whereas a value of 52.6% was observed in the control group. The proportion of *Actinobacteriota* and *Proteobacteria* was higher in the control group at values of 28.4% and 10.9%, respectively, whereas 12.6% and 0.8% were noticed in the AMA group (**Figure 3A**). At the genus level, the vaginal microbiota of the three groups mainly included *Lactobacillus*, *Bifidobacterium*, *Gardnerella*, *Streptococcus*, *Escherichia-Shigella*, and *Prevotella*, among others. *Lactobacillus* predominated in vaginal microbiota, and a lower proportion was observed in the control group (38.6%) than the AMA (81.2%) and NMA (81.8%) groups. The proportion of *Gardnerella* was higher in the control group (19%) than in the AMA group (5.1%) (**Figure 3B**). From the above two figures, we found that the composition of the vaginal microbiome in AMA and NMA groups was similar. However, the distribution in the control group was distinctly different.

The gut microbiota of the three groups was mostly made up of *Firmicutes*, *Bacteroidota*, *Fusobacteriota*, *Campilobacterota*, *Proteobacteria*, and *Actinobacteriota*. *Firmicutes* were still abundant in pregnant women, with a proportion of 55% in the AMA group and 51.9% in the NMA group, whereas a 35.3% proportion was recorded in the control group. The proportion of *Bacteroidota* was higher in the control group (47.9%), whereas 35.4% was observed in the AMA group (**Figure 3C**). At the genus level, the gut microbiota of the three groups mainly included *Bacteroides*, *Prevotella*, *Porphyromonas*, *Lactobacillus*, and *Fenollaria*, among others. *Prevotella* predominated in the vaginal microbiota, featuring a lower proportion in the control group (25.9%) than the AMA (19.7%) and NMA (16.8%%) groups. The proportion of *Bacteroides* was higher in the control group (16.1%) than in the AMA group (5.8%) (**Figure 3D**).

### Significant Changes in Abundance of Microbiota

**Figure 4:**
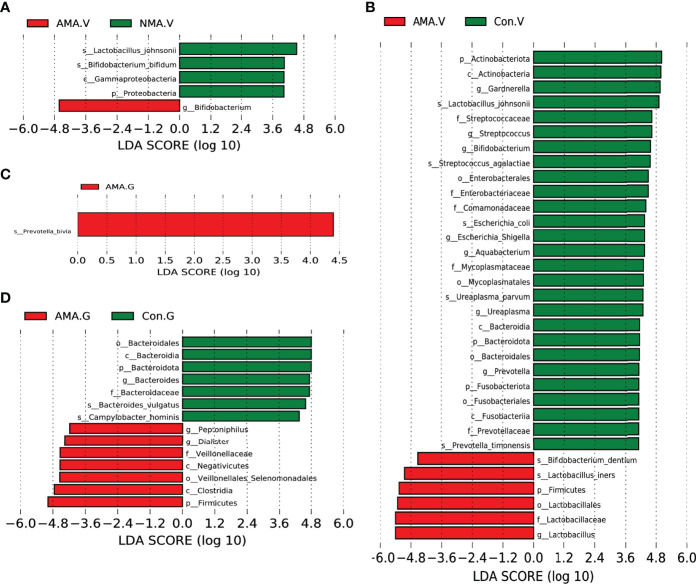
**(A)** LEfSe (LDA effect size) between AMA and NMA groups in vaginal microbiota. **(B)** LEfSe between AMA and control groups in vaginal microbiota. **(C)** LEfSe between AMA and NMA groups in gut microbiota. **(D)** LEfSe between AMA and control groups in gut microbiota.

We performed LEfSe analysis and linear discriminant analysis (LDA) to distinguish the three groups by identifying microbiota biomarkers at different taxonomic levels and estimating the effect size of each differentially abundant microbiota. This LDA score was set to 4. The larger the LDA score, the greater the effect of the microbiota had on the difference of the abundance between the comparative groups. Compared with the NMA group, the vaginal microbiota in the AMA group was mostly enriched with *Bifidobacterium.* However, *Proteobacteria*, *Bifidobacterium bifidum*, *Gammaproteobacteria*, and *Lactobacillus johnsonii* were significantly abundant in the NMA group (**Figure 4A**). Compared with the control group, the vaginal microbiota in the AMA group was mostly enriched with Firmicutes, Lactobacillales, Lactobacillaceae, *Lactobacillus*, *Lactobacillus iners*, and *Bifidobacterium dentium.* However, *Prevotella timonensis*, Bacteroidales, *Ureaplasma parvum*, Mycoplasmataceae, *Aquabacterium*, Fusabacteriales, Comamonadaceae, *Escherichia coli*, Enterobacteriaceae, *Streptococcus agalactiae*, *Bifidobacterium*, Actinobacteria, *Gardnerella*, and *Lactobacillus johnsonii* were significantly abundant in the control group (**Figure 4B**).

As for the gut microbiota in the three groups, the abundance of *Prevotella bivia* was significantly higher in the AMA group than in the NMA group (**Figure 4C**). The abundances of *Peptonophilus, Dialister, Veillonellaceae*, *Negativicutes*, *Clostridia*, *Firmicutes*, and *Veillonellales* (*Selenomonadales*) were significantly higher in the AMA group compared with the control group. However, the abundance of *Bacteroidota*, *Bacteroides vulgatus*, and *Gampylobacter hominis* was significantly higher in the control group than in the AMA group (**Figure 4D**).

## Discussion

Recent studies have shown possible links between microbiota and pregnancy complication (Guzzardi et al., 2022; Molina-Vega et al., 2022; Yeo et al., 2022). However, the relationship between microbiota and maternal age has been rarely reported. Therefore, we investigated the changes of vaginal and gut microbiota in women with AMA by using 16S rRNA sequencing.

In our study, we found that the vaginal microbiota in the three groups is mostly made up of *Firmicutes*, *Actinobacteriota*, *Proteobacteria*, and *Bacteroidota*, as shown in **Figure 3**. Similar to previous reports (Li et al., 2021), we observed that *Lactobacillus* was the dominant bacterium in the vagina of women in the three groups. The composition of the female genital tract microbiota is influenced by numerous factors, including age, pH in vagina, hormonal secretions, the menstrual cycle, contraceptives, antibiotic use, and sexual activity (Prince et al., 2015; Nasioudis et al., 2017). *Lactobacillus*, the dominant bacterium in the vagina of women during pregnancy (Dominguez-Bello et al., 2010; Ma et al., 2012), can bind to the surface of vaginal epithelial cells to prevent the attachment of other microorganisms in the vagina. It can not only produce lactic acid by decomposing glycogen in the vagina to maintain a stable pH, but also kill intracellular microorganisms by inducing the autophagy of vaginal epithelial cells (Witkin and Linhares, 2017). The stability and abundance of *Lactobacillus* species in the vaginal microbiota depend on hormone levels (Hay, 2005). In the present study, the abundance of *Lactobacillus* was the highest in the vagina of women in the three groups, as the yellow part shows in **Figures 3A, B**, especially in the AMA and NMA groups. This was consistent with Pacha-Herrera’s report earlier, in which a higher amount of *Lactobacillus* was found in pregnant women when compared to non-pregnant women (Pacha-Herrera et al., 2020). The abundance of *Lactobacillus* was markedly lower in the control group, and this finding was related to the increase in estrogen in women during pregnancy (Taddei et al., 2018). A high proportion of *Gardnerella* (19%), which is an anaerobic bacterium considered one of the etiological agents of bacterial vaginosis (Morrill et al., 2020), was observed in the control group. *Gardnerella* was the dominant bacterium in the vagina of women over 35 years old. This result of our research was consistent with that of Chen et al., (2021).

In our research, the alpha diversity (PD and ACE) of vaginal microbiota was significantly higher in the AMA group than in the NMA group (*p* < 0.05), which means that the number of operational taxonomic units was higher in the AMA group. Thus, we speculated that more miscellaneous bacteria were present in the vaginal microbiota of women with AMA. However, due to the limited sample size of the AMA group, our conjecture needs to be further verified. In our research, the abundance of *Bifidobacterium* was higher in the vaginal microbiota of the AMA group. Freitas and Hill (2017) observed that *Bifidobacterium* has a protective effect on the vagina of older women. This bacterium can also reduce the risk of preterm birth and improve the microecology of the vaginal microbiome (Tabatabaei et al., 2019). *L. johnsonii*, which is one of the most commonly detected strains in the vagina of reproductive women (Dobrut et al., 2018; Mehta et al., 2020), was significantly abundant in the NMA group in our study. This bacterium can not only inhibit the growth of pathogens (*Escherichia coli*, *Gardnerella vaginalis*, *Proteus mirabilis*, and *Candida albicans*) (Ahire et al., 2021) but also reduce the incidence of preterm birth (Tabatabaei et al., 2019). A significant drop of *Lactobacillus* was also observed in the women with aerobic vaginitis of different ages (Pacha-Herrera et al., 2020). Therefore, the abundance of *L. johnsonii* decreased significantly in the AMA group, and this result may be associated with the increased risk of complications during pregnancy in women with AMA. The beta diversity of vaginal microbiota in the AMA group significantly decreased compared with the control group (*p* < 0.001). This finding demonstrated the significant difference between women with AMA and non-pregnant women in terms of vaginal microbiota. Freitas et al. (2017) also reported that the vaginal microbiome of healthy pregnant women had reduced richness and diversity and less *Mycoplasma* and *Ureaplasma* sp. compared with non-pregnant women. The abundance of *Prevotella*, *Bacteroidales*, *Ureaplasma*, *Mycoplasmataceae*, *Streptococcus*, *Comamonadaceae*, *Escherichia*, and *Gardnerella* was significantly higher in the control group than in the AMA group. Most of these bacteria were anaerobic, the increased abundance of which was related to bacterial vaginosis, premature rupture of membranes, preterm birth, gestational diabetes mellitus, and preeclampsia (Fettweis et al., 2019; Kong et al., 2019; Liu et al., 2019; Lin et al., 2020; Mohamed et al., 2020; Sprong et al., 2020). *Gardnerella* and *Prevotella*, in particular, increase the risk of bacterial vaginosis from previous studies (Pacha-Herrera et al., 2020). It is well known that bacterial vaginosis in pregnancy is associated with an increased risk of preterm birth (Foessleitner et al., 2021). Therefore, the increased abundance of beneficial bacterium and the decrease in pathogenic bacterium and miscellaneous species may be a self-protective mechanism for pregnant women to reduce the incidence of infection and miscarriage.

In recent years, studies related to gut microbiota and pregnancy have been extensively focused. The gut microbiota, composed of trillions of symbiotic microorganisms, is essential for host’s health and survival (Fung et al., 2017). The gut microbiota changes along with modifications in hormone levels and immune metabolism during gestation (Gohir et al, 2015a). The microbial diversity in the gut at the start of pregnancy appears to be similar to that in nonpregnant women, and the abundance of gut bacteria associated with inflammatory states increases as pregnancy advances (Edwards et al., 2017). Zhang et al. (2021) used 16S rRNA sequencing to explore the longitudinal changes of gut microbiota in diabetic mice. They observed a trend toward increased diversity and richness from 6 to 26 weeks in mice. In the present study, we observed a trend toward decreased diversity and richness in the AMA group as indicated by the PD, ACE, and Shannon indices, also suggesting that aging is an important factor affecting microbial composition. Similar to Lee and Sanlibaba’s studies (Lee et al., 2022; Sanlibaba et al., 2022), the gut microbiota changes are generally characterized by the decrease in biodiversity and enrichment of opportunistic pathogens with aging. The gestational gut microbiota is characterized by a low alpha diversity index (intraindividual bacterial diversity) and a high beta diversity index (interindividual bacterial diversity) in the third trimester (Wallace et al., 2019; Mesa et al., 2020). We also found that the alpha diversity of the AMA group was slightly lower than that of the NMA group. The difference between the two groups was not statistically significant; it might be related to the small sample size of this study. *Firmicutes* and *Bacteroidota* are the most abundant gut microbiota in human beings (Ley, 2016). Liu et al. (2017) indicated that the gut microbiota of pregnant women was mostly made up of *Firmicutes*, *Bacteroidota*, *Fusobacteriota*, and *Proteobacteria* in South China. In addition, they observed that the abundance of Bacteroidota was higher than that of *Firmicutes* in the gut microbiota in the third trimester. However, the results of our study showed the contrary; the proportion of Bacteroidota was the highest in non-pregnant women. The microbiota undergoes changes according to age, represented by a human study showing that the core microbiota of elderly subjects was different from that of younger adults, with a higher proportion of *Bacteroides* species (Claesson et al., 2011). The results are consistent with ours, in which the proportion of *Bacteroides* was higher in the control group. *Prevotella bivia*, a kind of Gram-negative anaerobic bacillus, was enriched in the AMA group and a part of the human oral microbiome (Accetto and Avgustin, 2021). *Prevotella* species are more common in non-Westerners who consume a plant-rich diet (Wu et al., 2011). The association with a plant-rich diet has suggested that *Prevotella* is a beneficial microbe. They are dominant in the rumen and hindgut of cattle and sheep, where they facilitate the fermentation of protein and carbohydrate from foods. Consumption of dietary fiber improves glucose metabolism, which is associated with increased abundance of *Prevotella* (Kovatcheva-Datchary et al., 2015). However, *Prevotella* in the gut has also been linked to inflammatory conditions (Dillon et al., 2016). The reasons for the increased abundance of *Prevotella* of gut microbiota in women with AMA still need further research. *Peptonophilus*, *Dialister*, Veillonellaceae, Negativicutes, and Clostridia were concentrated in the AMA group. *Dialister* is enriched in the gut microbiota of patients with osteoporosis and may be a microbial marker of spondylarthritis disease (Xu et al., 2020). A decrease in blood pressure in rats was associated with the lowering of *Veillonellaceae*, which includes succinate-producing bacteria (Galla et al., 2020). The abundance of Veillonellaceae increased in the gut microbiota of mice with abnormal glucose metabolism (Cheng et al., 2018). A research in Pakistan showed that *Negativicutes* are enriched in the gut microbiota of obese type 2 diabetes mellitus patients (Ahmad et al., 2019). Therefore, the accumulation of these bacterium may be associated with the increased risk of gestational diabetes and gestational hypertension in AMA.

As far as we know, this research is the first study that analyzed the vaginal and gut microbiota of women with AMA. We observed that the alpha diversity of vaginal microbiota in women with AMA significantly increased. The beta diversity was significantly compared with that of non-pregnant older women. The presence of various statistical biomarkers in the AMA group suggests significant differences in the distribution of microbiota in women with AMA and NMA. However, this was only a preliminary research because the total quantity of the samples was limited. Although we have not found the kind of specific bacterium of women with AMA, the dysregulation in vaginal and gut microbiota of women with AMA was confirmed in our study. In the future, we would like to increase the sample size and detect the metabolic characteristics of the microbiome in women with AMA by metagenomic sequencing and to further find the type of specific bacteria from women with AMA and deeply explore the relationship between specific bacteria and adverse pregnancy outcomes in women with AMA.

## Data Availability Statement

The original contributions presented in the study are publicly available. These data can be found here: NCBI, PRJNA812337.

## Ethics Statement

The studies involving human participants were reviewed and approved by the Medical Ethical Committee of Zhujiang Hospital, Southern Medical University. The patients/participants provided their written informed consent to participate in this study.

## Author Contributions

YH: Writing—review and editing, methodology, and resources. DL: Writing—original draft, data curation, and formal analysis. WC: Formal analysis and writing—review and editing. HZ: Data curation and formal analysis. MIS: Resources and software. CL: Supervision and project administration. SP: Conceptualization, visualization, funding acquisition, and project administration. All authors contributed to the article and approved the submitted version.

## Funding

This work was funded by grants from the National Key R&D Program of China (2019YFC0121904).

## Conflict of Interest

The authors declare that the research was conducted in the absence of any commercial or financial relationships that could be construed as a potential conflict of interest.

## Publisher’s Note

All claims expressed in this article are solely those of the authors and do not necessarily represent those of their affiliated organizations, or those of the publisher, the editors and the reviewers. Any product that may be evaluated in this article, or claim that may be made by its manufacturer, is not guaranteed or endorsed by the publisher.
